# Prevalence and predictors of self-medication among university students in Ethiopia: a systematic review and meta-analysis

**DOI:** 10.1186/s40545-021-00391-y

**Published:** 2021-12-16

**Authors:** Getahun Fetensa, Tadesse Tolossa, Werku Etafa, Ginenus Fekadu

**Affiliations:** 1 Department of Nursing, School of Nursing and Midwifery, Institute of Health Sciences, Wollega University, Nekemte, Ethiopia; 2 Department of Public Health, Institute of Health Sciences, Wollega University, Nekemte, Ethiopia; 3 Department of Pharmacy, Institute of Health Sciences, Wollega University, Nekemte, Ethiopia; 4 Present Address: School of Pharmacy, Faculty of Medicine, The Chinese University of Hong Kong, N.T, Shatin, Hong Kong

**Keywords:** Ethiopia, Meta-analysis, Self-medication, Students, Systematic review

## Abstract

**Background:**

Self-medication of medicines is a global issue particularly among those with good access and familiarity with medications such as university students. It has a significant impact on drug resistance and medication-related complications. There are limited and inconsistent studies on self-medication practices in Ethiopia. The aim of this systematic review and meta-analysis was to estimate the pooled prevalence of self-medication and its predictors among university students in Ethiopia.

**Methods:**

A systematic review and meta-analysis was conducted to assess the prevalence and predictors of self-medication among university students in Ethiopia. Published articles from various electronic databases such as Medline, Hinari, Pub Med, Cochrane library, and the Web of Science were accessed. In addition, a manual search was performed including Google Scholar. Searching of articles were searched from January 1st to February 1_,_ 2021. All observational studies conducted among university students in English language were included in the review. Two reviewers independently assessed articles before inclusion in the final review using the Joanna Briggs Institute Meta-Analysis of Statistics Assessment and Review Instrument (JBI-MAStARI) instrument for critical appraisal. The *I*^2^ test was used to assess heterogeneity. Since the included studies exhibited high heterogeneity, a random-effects model was used to estimate the pooled prevalence of self-medication.

**Results:**

We found of 812 published and unpublished studies in our search. Finally, 31 full-text studies were reviewed, and 13 studies fulfilled the inclusion criteria and were included in the final meta-analysis. A total of 5377 study respondents from 13 studies were included in the study. The results of our study revealed that the pooled prevalence of self-medication among university students was 49.41% (95% CI 38.67%, 60.13%). The included studies had a sample size ranging from 250 to 792 with the lowest prevalence (19.87%) of self-medication from the University of Gondar, whereas the highest prevalence (77.01%) was recorded in a study conducted at Arsi University. From the pooled estimation, there was a significant association between self-medication and income (OR = 0.67: 95% CI 0.55–0.80). However, the association between self-medication and year of study and sex of participants was insignificant.

**Conclusion:**

The pooled prevalence of self-medication among Ethiopian university students was relatively high compared to the current global health problem with an increase in anti-microbial resistance. Health professionals and concerned bodies should pay attention to raising awareness regarding the consequences of using medications without prescription.

**Supplementary Information:**

The online version contains supplementary material available at 10.1186/s40545-021-00391-y.

## Background

Self-medication is the use of medicines by people based on their own interests without the prescription of health professionals. It includes the use of medicines, previously prescribed medicine for similar cases, and treatment of family members especially for minor cases and elderly populations. Self-medication is currently a global issue [1]. Based on socio-economic and socio-demographic factors; the types, extent, and reason for self-medication can vary from country to country along with its effect [2]. The source of information influences self-medication practice [3, 4]. Self-medication may arise from different factors that can be categorized as health professional and consumer factors. Some of the self-medicating groups complain that they are not comfortable with health professional behavior and do not have enough confidence in knowledge of health professionals while others need to practice participation in their health management [5]. Consumers perceive two different perceptions by consumers from the consumer’s perspective. The first perception of consumers agrees that self-medication is not safe, and the counter part is the opposite [1]. Some students also perceive self-medication as part of their self-care [6].

A significant portion of the students had self-prescribed a drug based on the previous doctor’s prescription for the same disease and their previous experience. The condition might rise from improper advice of the professional at last contact [7, 8]. Others also practiced self-medication either because their illness was not serious, or they had prior experience with the drug [9]. Most students obtain self-medication from pharmacies, which indicates the irresponsiveness of pharmacy professionals to give medications without a prescription [10]. Inappropriate self-medication may lead to a delay in the diagnosis of a serious health problem and its economic burden on the patient and the health-care system that predisposes patients to needless medicines. The problem might harm the health, lead to the side effects and adverse effects of home remedies and herbs, which might worsen the patient’s condition and the patients might suffer longer than necessary, and missed many workdays [11].

Self-medication practice among university students is related to factors such as like being female, being the undergraduate and increasing year of study to be practiced in high prevalence [2, 5, 12–16]. The prevalence of self-medication among health science students in India was 92.7% [13], in north India among nursing students 88.24% [17]. As these countries are culturally proximal, in Brazil among nursing students 76.0% [18], and 97.8% among medical students in Kuwait [19]. Additionally, in Iran university 65% [15], Eastern Saudi Arabia 90.5% [20], Coastal South India 78.6% [21], United Arab Emirates 86% [22], and Oman 94% [9].

Therefore, many factors were directly related to self-medication among university students. Of these illnesses like headache, menstrual discomfort, constipation, fever, cough, and abdominal discomfort were the common illnesses for which students were seeking self-medication [2, 19, 23–25]. Studying self-medication practices among university students is crucial because they are an educated part of the community, and it is believed that they can provide information regarding the consequences of self-medication for the general population outside the university. In another way appropriate self-medication behavior can have many benefits. For example, like increasing access to medication and relief for the patient, the active role of the patient in his or her own health care, better use of physicians and pharmacists’ skills and reduced (or at least optimized) burden of governments due to health expenditure linked to the treatment of minor health conditions. However, inappropriate self-medication behavior can cause adverse health outcomes. There are limited and inconsistent studies on self-medication practices in Ethiopia. Therefore, this study was aimed at estimating the pooled prevalence and identify factors contributing to self-medication in population groups assumed to experience frequent self-medication.

## Methods

### Search strategy

We checked for the presence of existing systematic reviews and meta-analyses on a similar topic using PROSPERO to avoid duplication at the beginning (CRD42018099975). Only published and all observational studies were considered. All potentially relevant studies in the databases Medline, Hinari, Scopes, PubMed CINAHL, PopLine, MedNar, Embase, Cochrane library, JBI Library, Web of Science and Google Scholar databases were included. We accessed all published studies since 2000 using the following search terms *‘Prevalence’ OR ‘proportion’ OR ‘magnitude’ OR ‘Epidemiology’ AND ‘self-medication’ OR ‘self-prescription’ AND ‘Ethiopian’ ANFD’ university’ OR’ College’*. A list of references from identified articles was also reviewed to identify additional articles.

Literature was downloaded and imported to Mendeley desktop reference citation to maintain and manage citation and facilitate the review process and the Preferred Reporting Items for Systematic Reviews and Meta-Analyses (PRISMA) guideline was used during the review process. The searching of articles was done from January 1st to February 1, 2021.

### Selection and eligibility criteria

This systematic review included studies that were conducted on the prevalence of self-medication and associated factors in Ethiopia and participants were university students. The review included all studies conducted in Ethiopia, all observational studies and studies published in the English language. We considered all published and unpublished studies that were published in the form of journal articles, reports, master thesis and dissertations. We excluded studies conducted outside university students. Conference abstracts and non-human studies were excluded. We tried to contact the primary authors of the articles with incomplete information, and we excluded articles that were not accessible after contacting the principal investigator two times via email.

### Study outcomes

This meta-analysis and systematic review considered two main outcomes. The primary outcome was to determine the prevalence of self-medication among university students. The second outcome of this study was to identify factors associated with self-medication. The prevalence was calculated by dividing the number of participants engaged in self-medication practice by the total number of participants who have been included in the study (sample size). For the second outcome, we determined factors associated with self-medication in the form of the odds ratio (OR). Three major factors were assessed by each primary study were selected to explore their association with self-medication. For each factor, we calculated the OR based on binary outcomes from the primary studies. The factors assessed for this review was sex (male versus female), income per month (< 500 versus > 501 Ethiopian birr) and year of study (≤ 2 years versus ≥ 3 years).

### Quality assessment and data extraction

The reference management software (Mendeley pdf organizer) was used to combine database search results and to remove duplicate articles manually. Two reviewers independently assessed articles before inclusion in the final review using the Joanna Briggs Institute Meta-Analysis of Statistics Assessment and Review Instrument (JBI-MAStARI) instrument for critical appraisal [26]. For the primary outcome, the data extraction format included the region in the country where the studies were conducted, the university where the studies were carried out, publication year, study design, sample size, response rate, and prevalence/practice of self-medication. For the second outcome (associated factors), the data extraction format was prepared for each specific associated factor. For each associated factor, to calculate the odds ratio, we were extracted the data from the primary studies in the form of two-by-two tables. Any disagreements between the authors during extractions were discussed and solved through consensus. Studies with a quality assessment score of six and above were included in the final review.

### Statistical analysis and synthesis

Before analysis, necessary data from each original study were extracted using Microsoft Excel spreadsheet form. The extracted data were imported to and analyzed using STATA version 14.1 statistical software. We generated the logarithm and standard error of the OR for each original study using “generate” command in STATA. We used Cochrane’s *Q* statistic (Chi-square), *I*^2^, and *p*-values to check for heterogeneity of the studies’ outcomes. Forest plots were used to present the results of the analysis and to visualize the presence of heterogeneity. Also point prevalence, as well as 95% confidence intervals, was presented in a forest plot format. From the result of the statistical test, significant heterogeneity was found among included studies (*I*^2^ = 99%, *p* < 0.001), and a random-effects meta-analysis model was performed to estimate the Der Simonian and Laird’s pooled effect. Besides, we conducted subgroup analysis (based on the region where studies were conducted) to identify the source of heterogeneity, and statistically significant results were declared in the presence of heterogeneity. A Funnel test was used to check publication bias. Further, we checked the statistical significance of publication bias using Egger tests. A *p*-value of less than 0.05 was used to declare the presence of publication bias.

## Results

### Selection and identification of studies

**Fig. 1:**
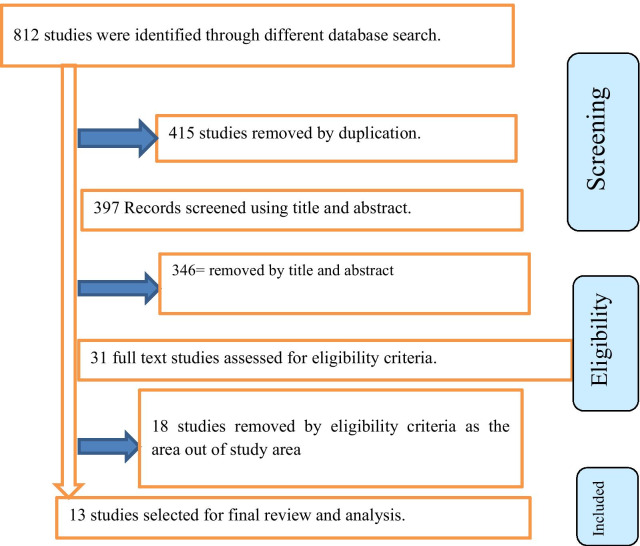
Flowchart describing the selection of studies for the systematic review and meta-analysis of prevalence and predictors of self-medication among university students in Ethiopia

We found a total of 812 published and unpublished studies throughout our search that were conducted in different regions of the country. Of the total identified, 415 duplicate records were removed, and 346 papers were excluded after screening by title and abstract. We assessed the full text of remaining the 31 studies for eligibility, of which 18 studies were excluded because they failed to meet the eligibility criteria. Finally, 13 studies were scored six and above on the JBI quality appraisal eligibility criteria and included in the meta-analysis. The Preferred Reporting Items for Systematic Reviews and Meta-Analyses (PRISMA) flow diagram to present the systematic review is shown in Fig. 1.

### Description of included studies

The 13 included studies were cross-sectional study design and published 2010 to 2020. In the current meta-analysis, 5377 study participants were involved to determine the pooled prevalence of self-medication among university students. Study participants were recruited from both sex having health and non-health backgrounds, the studies were adequately addressed the total population of catchment area to select the sample size as they have used appropriate sampling method. Accordingly, the minimum sample size was 250 [27] and the maximum was 772 [28]. The lowest prevalence (19.8%) of self-medication was reported in studies conducted in Gondar University, Amhara region [29], whereas the highest prevalence (77.01%) was reported in a study conducted in Arsi University, Oromia region [30]. In the present meta-analysis, eight Ethiopian Universities were represented. Eight of the studies were from Amhara [27–29, 31–34, 38], three from Oromia [30, 35, 36], and two from Tigray [24, 37]. Regarding the response rate, almost all studies had a response rate of greater than 90% (Table 1).

**Table 1:** Descriptive summary of studies included in the meta-analysis of the prevalence and determinants of self-medication among university students in Ethiopia

Authors	Year published	Region	University	Study design	Sampling size	Prevalence (%)	Response rate (%)	Newcastle Ottawa scale (10 pts)
Abay and Amelo [29]	2010	Amhara	Gondar	Cross-sectional	414	19.80	Not stated	7
Girma [37]	2011	Tigrai	Mekelle	Cross-sectional	283	43.24	92.20%	7
Mulugeta and Nasir [35]	2012	Oromia	Jimma	Cross-sectional	403	45.89	100	8
Tadele [24]	2014	Tigrai	Mekelle	Cross-sectional	407	44.50	96.4	8
Shimelis [30]	2016	Oromia	Arsi	Cross-sectional	548	77.10	95.3	8
Dessalegn and Gashaw [38]	2017	Amhara	Gondar	Cross-sectional	404	35.30	95.3	7
Abebe [27]	2017	Amhara	Debre markos	Cross-sectional	250	58.4	90.3	7
Dessalegn [31]	2017	Amhara	Gondar	Cross-sectional	404	32.70	95.3	7
Alemseged [36]	2017	Oromia	Riftvalley	Cross-sectional	400	72.8	90.2	7
Abebe et al. [32]	2020	Amhara	Amhara teaching college	Cross-sectional	344	68.02	92.5	7
Zelalem et al. [33]	2020	Amhara	Gondar	Cross-sectional	425	62.35	100	8
Nuhamin et al. [28]	2020	Amhara	Gondar	Cross-sectional	792	52.39	98.8	10
Segenet et al. [34]	2020	Amhara	Wollo	Cross-sectional	341	48.97	92.6	10

### Reason for self-medication, recall period, rational for the use of self-medication and most used drugs

Among the common illness and symptoms reported by students, headache was the most predominant reason with 25.8–69.1% across all selected studies with recall period from 2 to 6 months of duration. The drug groups range from antibiotics type, analgesics, anti-acids, and vitamins (Table 2).

**Table 2:** Common illnesses, medications used, reason for self -medication and recall period systemic review and meta-analysis among university students in Ethiopia

Authors	Common illness and symptoms	Medications used	Reason for selection of self-medication	Recall period
Abay and Amelo [29]	Fever and headache (25.8%) Cough and common cold (23.9%) Gastric pain (13.2%) Diarrhea (8.9%) Fever and chills (6.1%) Cough and chest pain (6.1%) Constipation (5.6%) Eye disease (3.8%) Other (stress, fatigue, loss of appetite) (6.5%)	Analgesics constituting (24.4%) Antibiotics (4.8%) Antimalarial (3.7%)	NR	2 months
Dessalegn and Gashaw [38]	NR	Antibiotics (59.9%) Analgesics or antipyretics (47.8%) Gastrointestinal drugs (28.8%) Respiratory tract infection drugs (24.7%), vitamins (22.1%) ORS (16.75)	NR	6 months
Shimelis [30]	Headache/fever (56.5%) Gastrointestinal disease (34.1%) Respiratory tract infection (31.8%) Eye disease (22.4%) Skin diseases or injury (17.45) Sexually transmitted disease (10.4%) Maternal/menstrual (29.2%)	NR	Disease is not serious (44.1%) Poor quality of service (27.1%) Emergency use (24.7%) Prior experience (23.4%) Took pharmacology course (21.1%) Saves time (20.3%) Less expensive (19.4%)	3 months
Tadele [24]	Cough common cold (43.85) Headache (34.8%) Abdominal pain (32.1%) Fever (24.1%) Diarrhea (19.6%) Eye infection (15.2) Nasal congestion (14.3) Tooth ache (12.55) Sore throat (12.5%) Others (17.9%)	Analgesics (44.4%) Antibiotics (42.7%) Topical applications (antifungal, anti-microbial, antihistamines and analgesics) (15.3%) Corticosteroids (9.7%) Others (3.6%)	Prior experience (69.6%), Minor illness (43.8%), Avoiding waiting time (36.6%), Cost-effectiveness (32. 1%), Others (21.4%)	3 months
Abebe [27]	Pain (head, body, tooth 60 (41%) Diarrhea 24 (16.4%) Fever 17 (11.6%) Nausea and vomiting 14 (9.6%) Dysmenorrhea 11 (7.5%), 10 (6.8%) Cough and itching 10 (6.8%)	Analgesics (52.7%) Antifungals (2.1%)	NR	
Dessalegn [31]	Headache (69.1%) Common cold (15.9%) Fever (15.9%) Abdominal discomfort (15.1%) Appetite loss (7.9%) Nausea and vomiting (7.1%) Heart burn/gastritis (6.3%) Diarrhea (5.6%) Impotence (5.6%) Contraception (4.8%) Eye disease (4%) Skin condition (0.8%)	Analgesics (53.2%) Antimicrobials (39.7%) Antacids (10.3%) Vitamins (8.7%)	Mildness of the disease (32.5%) Suggestions of friends (26.2%) Inexpensiveness of the practice (25.4%) Previous experience (19.8%) Do not trust health professionals (15.9%) Obtaining drugs easily (15.9%) Being embarrassed to talk about disease (7.9%) Long waiting time (7.1%) Long distance from health facility (3.2%) Can afford cost of drugs (2.4%)	6 months
Alemseged [36]	Fever and headache (69.3%) Gastric pain (67.5%) Cough and common cold (46.3%) Cough and chest pain (46%) Constipation (30%) Vomiting and diarrhea (29.5%) Fever and chills (23.8%) Skin symptoms (15.8%) Ear symptoms (15.5%) Others (7%)	Paracetamol (92%) Antacid (71.8%) Antibiotics (66.8%) NSAIDS (46.8%) Vitamins (285)	Non-seriousness of illness (81.3%) Quick relief (70.3%) Emergency use (45.8%) Prior experience (38.5%)	NR
Girma [37]	Headache or mild pain (47.3%) GI problems (30.8%) Eye and ear symptoms (29.1%) Vomiting (6.3%)	Antibiotic (47%) Pain killer (37%) Vitamins and minerals (10%) Other (2%)	Prior experience (39.10%) Mildness of illness (37.5%) Long waiting time (15.63%) Less costly (4.69%) Lack of interest in medical service (1.56%) Others (4.69%)	NR
Mulugeta and Nasir [35]	Headache 35 (36.85%) Abdominal pain 29 (30.55%) Cough 33 (23.16%) Fever (6.32%) Other (3.12%)	Analgesics 40 (49.38%) Antibiotics 29 (35.80%)	Illness (46.32%) Minor illness (25.26%) Time saving (24.16%) Low cost (4.21%)	NR
Abebe et al. [32]	Headache (32.1%) Abdominal pain (22.6%) Common cold (19.7%) Fever (15.8%) Cough (12.4%) Diarrhea (9.4%) Gastritis (6.8%) Skin problem (5.6%) Dysmenorrhea (4.3%) Others (3.9%)	NR	The similarity of illness with previous illness (38.9%) Disease not serious (24.4%) Easily obtaining drugs (17.1%) Friends’ suggestion (12.8%) Self-medication being cheaper (12.4%) Long waiting time in health service (9.8%) Long distance to the health facility (9.4%) Being embarrassed to talk about the disease (8.1%) Affordability of the cost of drugs (6.0%) Not trusting health professionals (4.3%) Others (3.4%)	NR
Zelalem et al. [33]	Headache (42.7%) Cough and common cold (21.1%) Pain (epigastric, tooth, body) (21.1%) Diarrhea (6.1%) Dysmenorrhea (3.4%) Other medical conditions ∗ (5.3%)	Analgesics (58.5%) Antacids (3.8%) Antispasmodics (9.1%) Antibiotics (26.5%) Others (2.3%)	Shortage of money (19.6%) Shortage of time (4.9%) Do not like visiting and getting checked up (10.2%) Mildness of illness (24.5%) An already known disease (39.6%) Other reasons (1.1%)	NR
Segenet et al. [34]	Headache (47.9%) GI infection (44.31%) Respiratory tract infection (28.74%) Pain (9.58%) Malaria (7.1%) Skin disease (3.59%) Eye disease (3.59%) Others (2.39%)	Analgesics (56.28%) Antibiotics (35.9%) GIT drugs (13.1%) Antimalarial drugs (7.18%) Anthelmintic drugs (6.58%) Anti-inflammatory drugs (4.19%) Supplements (4.19%)	Mildness of the illness (34.13%) Dissatisfaction with health-care service (26.34%) Familiarity with medicines and ailments (11.97%) Sufficient knowledge about medications (9.58%) Needed quick relief (9.58%) To save money (5.38%) To save time (2.99%) Other (2.99%)	NR
Nuhamin et al. [28]	Headache (86.5%) Respiratory tract infection (53%) Gastrointestinal disease (47.9%) Fever (38.7%) Eye disease (19.7%) Skin disease (18.3%) Maternal (19.5%) Others (1.2%)	Analgesic/antipyretic (71.8%) Antimicrobial (57.3%) Respiratory drugs (32.7%) Gastrointestinal drugs (29.1%) Vitamins (12%) Oral rehydration salt (7.9%) Others (1.4%)	Disease is not serious (76.1%) Emergency use (59%) Prior experience about the drug (own and/or friends, read about it, etc.) (25.3%) Less expensive in terms of time/money (25.3%) For prevention of known/unknown disease(s) (20.3%)	NR

*NR* not recalled

### Prevalence of self-medication

**Fig. 2:**
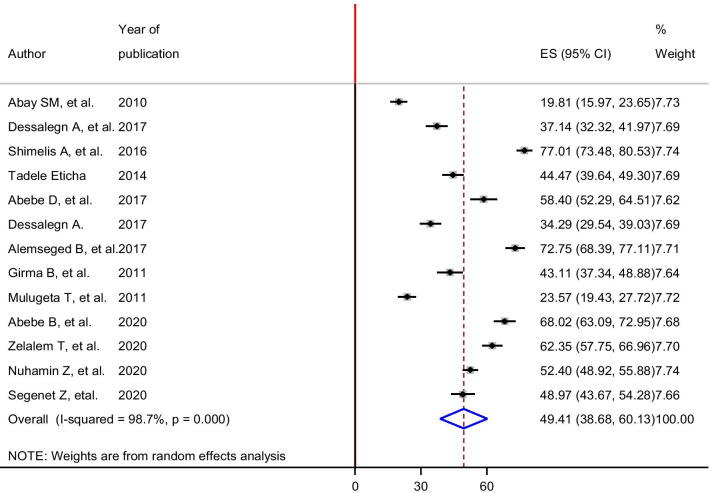
Forest plot of the pooled prevalence of self-medication among university students in Ethiopia

**Fig. 3:**
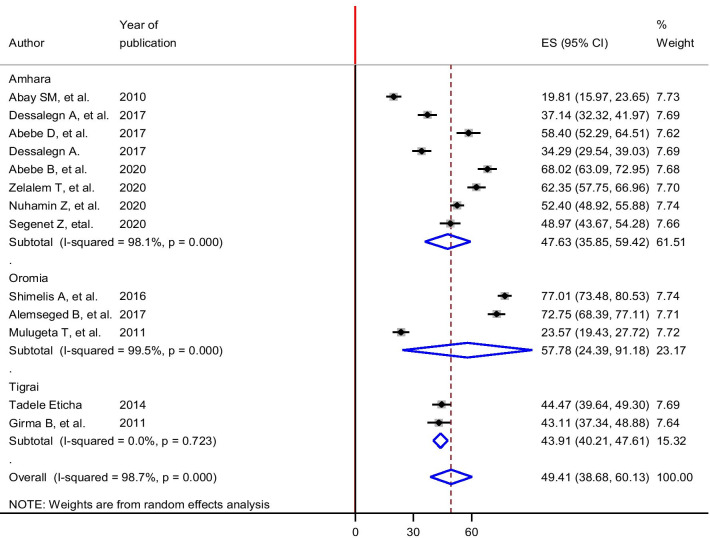
The pooled estimated prevalence of self-medication among university students by region among in Ethiopia

High heterogeneity was observed across the included studies (*I*^2^ = 98.7%, *p* < 0.001). Therefore, a random-effects meta-analysis model was executed to estimate the pooled prevalence of self-medication among university students in Ethiopia. The 13 included studies revealed that a pooled prevalence of self-medication among university students in Ethiopia was 49.40% with 95% confidence interval of 38.67–60.13% (Fig. 2). In this meta-analysis, we computed subgroup analysis for the prevalence of self-medication based on the region where the study was conducted. Accordingly, the prevalence ranged from 43.91% in the Tigray region to 57.78% in the Oromia region (Fig. 3).

### Publication bias

**Fig. 4:**
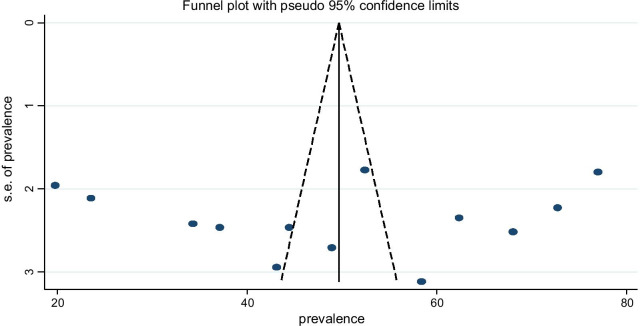
Funnel plot with 95% confidence limits of the pooled prevalence of self-medication among university students in Ethiopia

To assess the presence of publication bias, funnel plot, and Egger test at 5% significant level was performed. The funnel plot was asymmetry, but Egger and Begg’s tests showed that there was no statistically significant presence of publication bias with (*p*-value = 0.834, 0.855), respectively (Fig. 4).

**Fig. 5:**
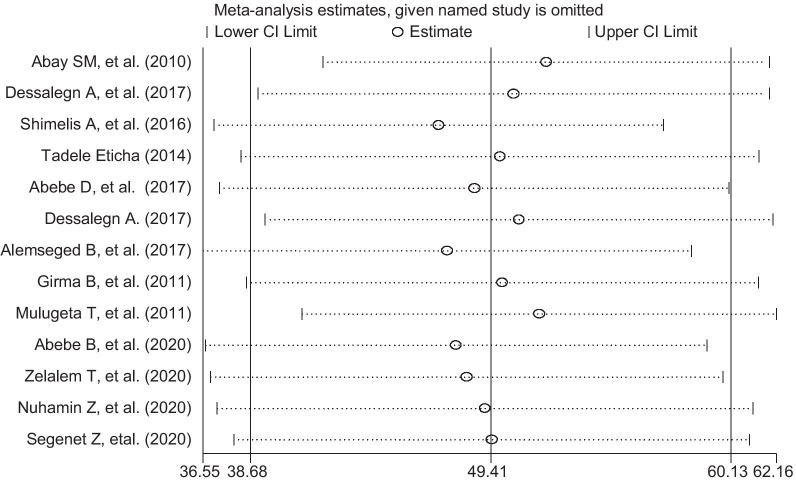
Sensitivity analysis for single study influence of self-medication prevalence in Ethiopia

To identify single study influence on the overall meta-analysis, a sensitivity analysis was performed using a random-effects model and the result showed there was no strong evidence for the effect of single study influence on the overall meta-analysis (Fig. 5).

### Predictors of self-medication

#### Association between sex and self-medication

**Fig. 6:**
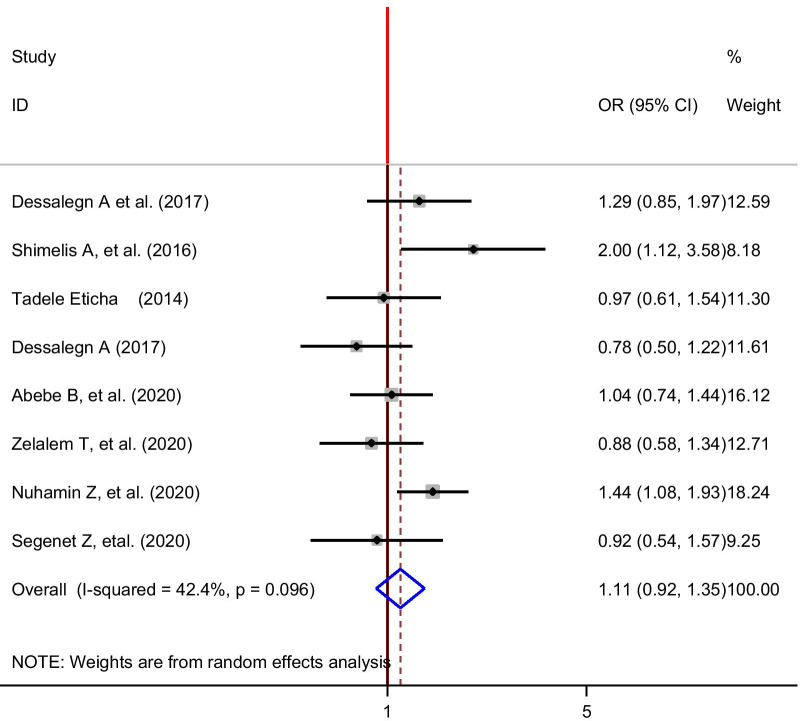
The pooled odds ratio of the association between sex and self-medication among university students in Ethiopia

To show the association between self-medication and sex of patients, eight studies [24, 28, 30–34, 38] were selected for meta-analysis. Two studies [28, 30] showed, there was a statistically significant association between self-medication and sex and six studies showed that there was no significant association between medication self-practice and sex of the respondents. The pooled finding of the analysis showed that there was no significant association between medication self-practice and the sex of the students. The random effect model was computed due to moderate heterogeneity (*I*^2^ = 42.4, *p* = 0.096) (Fig. 6).

#### Association between self-medication and year of study

**Fig. 7:**
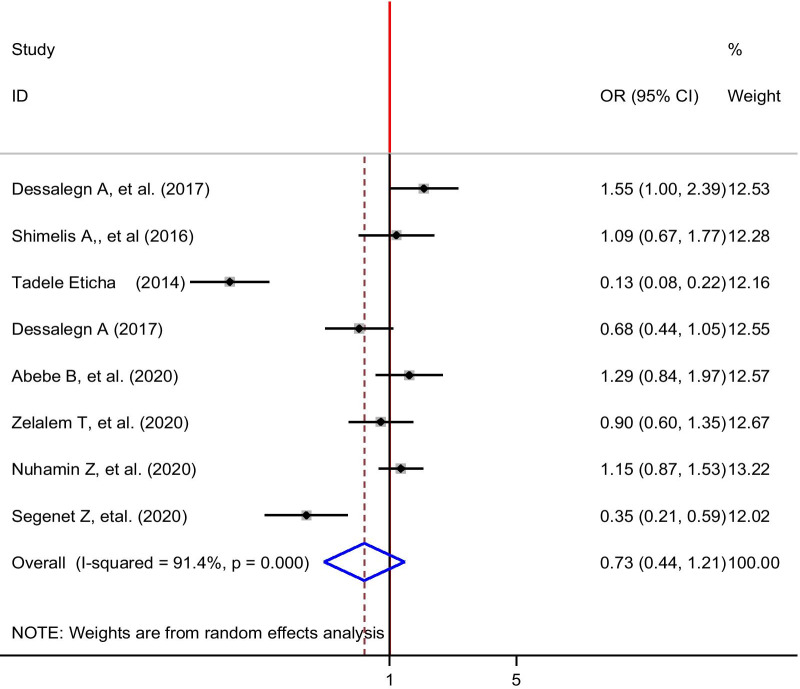
The pooled odds ratio of the association between year of study and self-medication among university students in Ethiopia

Eight studies [24, 28, 30–34, 38] were included to determine the association between self-medication and year of study. Two studies [24, 34] showed a statistically significant association between self-medication and year of study, while five studies showed no statistical significance between self-medication and year of study [28, 31–33, 38]. The pooled findings from these eight studies revealed that self-medication was not significantly associated with the year of study (OR: 0.73, 95% CI 0.44, 1.21) (Fig. 7).

#### Association between self-medication and income

**Fig. 8:**
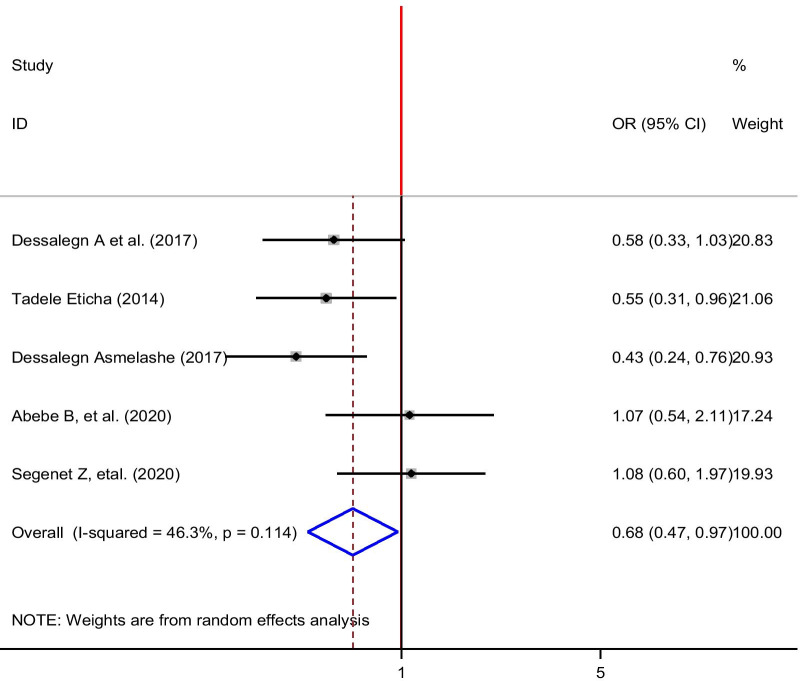
The pooled odds ratio of the association between income level and self-medication among university students in Ethiopia

Five articles were included to examine the association between self-medication and income [24, 31, 32, 34, 38]. From those involved, two studies showed a significant association between income and self-medication [24, 38]. From pooled estimation, there was a significant association between self-medication and income. Also, the test showed that there was heterogeneity between the included studies (*I*^2^ = 46.3% and *p* = 0.111) which suggests random-effects, meta-analysis model. Students who had income less than 500 Ethiopian birr was 32% less likely to participate in self-medication as compared to the student who had income greater than 500 Ethiopian birr (OR: 0.68: 95% CI 0.47, 0.97) (Fig. 8).

## Discussion

This systematic review and meta-analysis including 13 studies published between 2010 and 2020 were included to assess the pooled prevalence of self-medication and factors associated with self-medication among university students in Ethiopia. As far as our knowledge is concerned, this meta-analysis was the first of its kind in Ethiopia to estimate the pooled prevalence and predictors of self-medication practice among university students in the country. The result of our study revealed that a pooled prevalence of self-medication among university was 49.40% (95%: 38.67–60.13%). This indicated that access to drugs around university and knowledge of illness and treatment choice remains the fundamental contributor to self-medication practice among university students [10]. In another it can be taken as good opportunity in which increased access to medication and relief for the patient, by increasing patient self-care practice minimize time for seeking health care, better use of physicians and pharmacists’ skills and reduced burden of governments due to health expenditure linked to the treatment of minor health conditions. But irrational use of self-medication behavior can result in adverse health outcomes, such as taking antibiotics for colds. Additionally, the higher practice might be due to it is cheap and no medical appointment [18]. This finding was consistent with the study conducted on Chinese students [16] and Uganda [4]. However, our study revealed lower self-medication prevalence than other studies [12, 13, 15, 17, 18, 21, 39–46]. The result also was slightly higher than the survey report from Palestine 33.9% [47]. Here the difference might be participants from Palestine use herbal medicine for self-medication while a participant of this study uses all categories of drugs. The prevalence of self-medication can vary from place to place, which might be due to study methodological differences and variation in socio-demographic characteristics of the study participants.

The result of the study revealed that self-medication was not significantly associated with the year of study (OR: 0.79, 95% CI 0.43, 1.35). The random effect meta-analysis model indicated that there was no association between sex and self-medication in four studies [30, 31, 37, 39] that fulfill inclusion criteria. The result was contrary to the study conducted in Serbia [42], Uganda [4], sub-Saharan Africa [49], and India [13]. In these studies [24, 29, 30, 44–51] respondents had used antibiotics ranging from 4.8% [40] to 66.7% [51] without prescription. The result was in line with the study’s finding from India, which was 63.91% [49], Brazil ranged 11.1–55.6% [18], Iran 34.6% [15] and India 39.3% [21]. It is fact that irrational use of antibiotics leads to substantial adverse drug reactions, antibiotic resistance, treatment failure, and drug-related toxicity.

In another way, the result from this study illustrated that drugs named analgesics/painkillers/anti-pain were used in the range of 24.4% to 53.2%. The result was slightly lower than study result from Oman 96.6% among males and 95.1% among females [9], in India 74.8% [21], Brazil 63.2% [18], and Saudi Arabia (55.4%) [46]. Analgesics were the most common class of medications used in self-medication practices, because of such drugs are used to treat simple common illnesses like headache, fever, and pain. This was due to many analgesics are OTC drugs that are by law obtainable without prescriptions.

Among nine studies fulfilled inclusion criteria, 7 of the reported that minor/non-seriousness of the disease was one of the reasons for self-medication practice [24, 27, 30, 35–39] with minimum incidence 25.6% [35] and maximum 81.3% [37]. The low severity of symptoms of illness was also frequently reported reason in line with study results from Egypt [50] and Belgium [45]. Here it can be justified that some respondents might look at their illness carelessness. In fact, this all factors can be controlled or managed with appropriate monitoring and evaluation using the result of this study.

The most common illness for self-medication was headache among different studies including coastal South India (64.7%) [21], Brazil [18] and Iran 85.5% [15]. Five of studies fulfilling inclusion criteria reported that prior experience/from previous experience of the disease was one of the reasons for self-medication [24, 30, 35–39] ranging from 19.8% [39] to 69.6% [24]. The finding was comparable with study result from Egypt 58.9% [50] and systemic reviews from developing countries [51].

Timesaving was one of the other reasons for consuming self-medication among study respondents [24, 30, 37, 39]. The result was consistent with the studies from Brazil [21], Belgium [45] and a systemic review of developing country [51]. Cost-effectiveness [24, 30, 35–37] was the common reason for some of the students to participate in these studies. It was similar with studies results from Belgium 4.37% [45] and a systematic review in developing the countries in Africa [51].

Evidence suggests that self-medication results in wastage of resources, increased resistance of pathogens and causes serious adverse health outcomes like adverse drug reactions and interactions. In another way, it has potential to hide signs and symptoms that result in harder to reach the correct diagnosis and can facilitate the occurrence of further infectious diseases. As well, the incorrect anti-microbial or wrong dosage usually seen in self-medication can lead to microbial resistance, treatment failure, and increased cost for health care [52–55]*.*

It is assumed that legislation might reduce consumption of self-medication, however result from Saudi Arabia suggested that the enforcement of the new legislation on the prohibition of selling antibiotics without a prescription to prevalence self-medication was high. We can imagine that legal enforcement alone cannot reduce self-medication, rather there should be the need to raise public awareness regarding the safe use of medications [55]. Furthermore, result within our study varies from different study result that might be due to differences in primary studies and comparison group.

As strength of the study, comprehensive search strategies were used in the current systematic review and meta-analysis. A random-effects model was used to address the potential variability across studies. As a weakness, the study was based only on the study conducted in the English language, limiting data from other languages. Additionally, only published studies were accessed, and we have only considered the studies published between 2010 and 2020, which may limit the variation of data. The result was limited to students only, which might be difficult to generalize for other stakeholders. Currently, Ethiopia has ten national regional state however, the studies were only within the three of the national state in the country in which we cannot talk about generalization.

## Conclusion

Prevalence of self-medication among Ethiopian university students was 49.4%, which is very high with the current global health problem with anti-microbial resistance increment and other toxicities. This problem was increased due to the easy availability of drugs for self-medication. In addition, the association between self-medication and year of study and sex of participants were insignificant, but statistically significant with income level.

We strongly recommend pharmacy vendors to avoid selling the drug without prescription and health professional for counseling on drug usage during ordering drugs. We want to extend our recommendation to policymakers for supervision and control in drug vendors’ strategic guidance. Health professionals and concerned bodies should give attention in raising awareness regarding the consequences of using of medications without prescription for university students.

## Supplementary Information

**Additional file 1. **PRISMA checklist.

**Additional file 2. **Newcastle data quality Assessment.

## Data Availability

Data and all materials of the manuscript are with the primary author and available at any time on request.

## Abbreviations

CI: Confidence interval
df: Degree of freedom
NR: Not reported
OR: Odds ratio
PRISMA: Preferred Reporting Items of Systematic Reviews and Meta-Analysis
PROSPERO: Prospective register of systematic reviews
